# Satisfaction With the Use of Subdermal Contraceptive Implant in Women Attending the Specialized Polyclinic Primary Health Care Center in Jeddah City: A Cross-Sectional Study

**DOI:** 10.7759/cureus.35902

**Published:** 2023-03-08

**Authors:** Razaz Wali, Abdulkarim M Alghamdi, Samer T Ahmed, Abdulaziz M Gammash, Mohammed M Bukhari, Khozam F Alkhozam, Mouath H Asiri

**Affiliations:** 1 Family Medicine, King Abdulaziz Medical City, Ministry of National Guard Health Affairs, Jeddah, SAU; 2 College of Medicine, King Saud Bin Abdulaziz University for Health Sciences, Jeddah, SAU; 3 Research Office, King Abdullah International Medical Research Center, Jeddah, SAU

**Keywords:** long-term contraceptive, patient satisfaction, contraception, subdermal implant, implanon

## Abstract

Background

Subdermal contraceptive implants are a convenient method of contraception for many women due to the ease of insertion and removal and because they require less follow-up with their health facility. In addition to the contraceptive benefits, women's satisfaction with such devices is essential, as this can affect their quality of life. This study aims to measure women's satisfaction with the subdermal contraceptive implant, Implanon® (Organon & Co., Jersey City, New Jersey, United States), its main side effects, and reasons for removal.

Methods

A cross-sectional study was conducted on women between the ages of 19 and 65 years who visited the Family Planning Clinic at the Specialized Polyclinic Primary Health Care Center in Jeddah, Saudi Arabia, between January 2018 and December 2021. An online questionnaire was distributed to the women who had Implanon inserted and 84 responded. Demographic and contraceptive data were collected, including the dates of insertion and removal of Implanon and side effects experienced while on Implanon.

Results

Of the 84 women, 65.84% were satisfied with Implanon, while only 19.04% were unsatisfied with the implant. The most common side effect reported was weight gain (54.76%), followed by menstrual Irregularity. (39.29%). The most common reason for removal was the end of the implant’s contraceptive duration (42.86%).

Conclusion

Most of the women treated at this primary healthcare clinic were satisfied with Implanon. In addition, most of the women removed the implant only due to its reaching the end of its contraceptive duration despite experiencing side effects, and most women said that they would recommend it to their family and friends.

## Introduction

The contraceptive implant Implanon® (Organon & Co., Jersey City, New Jersey, United States) is a reversible long-term etonogestrel-releasing device with a single rod that is inserted subdermally [1]. This contraceptive device was approved by the United States Food and Drug Administration in 2006 and has shown efficacy for three years [2]. It works by reducing fertilization primarily by inhibiting the release of luteinizing hormone, a hormone that promotes ovulation [3]. Implanon is inserted 6-8 cm above the elbow of the woman's non-dominant hand by an attending physician at the outpatient clinic [4]. It is a progestin-only contraceptive, which can be used by any female. Moreover, it can be used for women who have contraindications for estrogen-containing contraceptives [5]. Contraindications for using subdermal implants include liver diseases or tumors, venous thromboembolism, and pulmonary embolism [6]. In a systematic review of subdermal etonogestrel implants, five studies reported Pearl Indices that ranged between 0 to 1.4, which indicates that etonogestrel implants are an effective method of contraception. Implanon has shown good acceptance and continuation rates if women are appropriately counseled before administration [7,8].

Implanon is more convenient than oral or barrier contraceptive methods since no additional action is needed for three years after it is placed [6]. Other advantages include its cost-effectiveness and the quick return of fertility after removal [9]. However, Implanon has been documented to cause side effects, including amenorrhea and bleeding irregularities, which are considered Implanon's most prevalent side effects [5]. Other side effects include headache, weight gain, acne, and breast pain. All-cause side effects lead 13.6% of women to discontinue the use of Implanon [10]. These side effects are associated with an overall reduction in satisfaction [11]. In a study conducted in Eastern Turkey between 2004 and 2005, an examination performed six months after Implanon insertion showed that symptoms of dizziness, headaches, and nausea were present in 46%, 39%, and 29% of the recipients, respectively [12]. Furthermore, the study results showed that 92% of participants reported changes in the frequency or duration of their menstrual cycle in the first year of the study, with 9% of participants removing Implanon during the first year for this reason [12].

General satisfaction with the subdermal contraceptive Implanon has been studied in multiple countries. In Upper Egypt, a study was conducted on 304 women with a median age of 32 [13]. The study found that prolonged contraception was the most common reason for choosing Implanon (39.5%), while the most common reason for discontinuing Implanon was experiencing side effects, mainly menstrual disturbances [13]. In a retrospective 12-center study conducted in Switzerland, 991 women using Implanon who had at least one follow-up visit were enrolled to assess satisfaction, side effects, bleeding patterns, and duration of use [8]. Of these 991 women, only 11% reported normal bleeding, while 28% reported infrequent bleeding and 33% reported no bleeding. Additionally, when asked about satisfaction, 54% reported that they were very satisfied, 25% were satisfied, 15% were somewhat satisfied, and 6% were dissatisfied [8]. Moreover, 235 women (23.7% of the 991 women) removed the Implanon implant before its intended duration of use was reached [8]. Approximately 20% of women removed the implant due to side effects, with the most common side effect being prolonged and frequent bleeding (45%) [8]. In research conducted on multiple contraceptive methods in St. Louis, Missouri (United States), the 493 women who chose the implant reported a satisfaction rate of 79% [14]. Thus, these studies from Switzerland and the United States demonstrate that etonogestrel implants have a high satisfaction rate.

In Saudi Arabia, family planning services are accessible to all Saudi nationals free of charge. Further, most contraceptive options are available, whether they are provided by the government or the private sector. However, the Ministry of National Guard Health Affairs (MNGHA) provides free healthcare services only to its employees and their families. Implanon was recently added to the list of contraceptives available at public healthcare facilities of the MNGHA, which includes the Specialized Polyclinic Primary Health Care Center (SPC-PHC), the only center that provides Implanon in Jeddah city. However, there is a gap in information about women’s satisfaction with Implanon use, as well as its most common side effects in Saudi Arabia.

This study aims to assess the satisfaction of women who used the subdermal contraceptive implant Implanon and were treated at the SPC-PHC of MNGHA in Jeddah, Saudi Arabia, from 2018 to 2021. The study is also intended to identify the main side effects of Implanon and reasons for removal if it occurred.

## Materials and methods

Study design

This is a cross-sectional study of women who used subdermal contraceptive implants and were treated at the SPC-PHC of MNGHA from 2018 to 2021. The primary data collection tool was a survey that was distributed using mobile text messages. The survey assessed specific reasons for the removal of Implanon, various aspects of women's satisfaction while using the contraceptive, and the side effects experienced.

Identification of study participants

The study was conducted among women of childbearing age who used Implanon as a contraceptive method during 2018-2021. The included participants were MNGHA personnel or their family members. Women who had used contraceptives besides Implanon and those with hormonal diseases or imbalances during the study period were excluded. The target sample size of 154 was calculated using Raosoft software (Raosoft Inc., Seattle, Washington, United States) with a 95% confidence interval, a response distribution of 50%, a margin of error of ± 5%, and a statistical power of 80% [15].

Data collection

The primary data collection tool was the Implanon Removal Questionnaire adapted from a study conducted in KwaZulu-Natal, South Africa [16]. After obtaining approval from the corresponding author of this previous study, the questionnaire, which was originally in English, was translated into Arabic by a professional translator and reviewed for reliability by the research team. A self-administered questionnaire using Google Forms (Google LLC, Mountain View, California, United States) was sent via mobile text message to all the women who had Implanon inserted at the SPC-PHC of MNGHA from 2018 to 2021 through their phone numbers extracted from their medical records. Notably, both in-person and telephone interviews were forbidden by MNGHA during this study due to patient privacy issues. This study was approved by King Abdullah International Medical Research Center with study number SP21J/115/03. A consent form was embedded within the distributed questionnaire.

Data analysis

Data were collected using Google Forms and analyzed using JMP Pro software version 15 (JMP Statistical Discovery LLC, Cary, North Carolina, United States). For descriptive analysis, categorical variables were presented as frequency and percentage and numerical variables were presented as mean, standard deviation, median, and range. The graphical illustration for the categorical variables was in simple bar charts and tables.

## Results

The self-administered survey was sent to 254 women who used Implanon between 2018 and 2021. Only 84 respondents completed the survey. Their median age was 28 years (range 19-65). Eighty-one (96.43%) women were married and living with their husbands, and 52 (61.90%) had completed university. Sixty-four (79.01%) had had zero to three pregnancies and 49 (58.33%) planned to have children in the future. Moreover, 42 (50.00%) women had previously used oral contraceptives, and 34 (40.48%) had previously used Implanon. Eight (9.52%) women had never used any contraceptive method earlier (Table 1).

**Table 1:** Characteristics and reproductive history of study participants

Participants’ characteristics	
Age, years, n (%) (n=84)	
18-24	16 (19.05)
25-30	34 (40.48)
31-35	19 (22.62)
36-40	9 (10.71)
>40	6 (7.14)
Highest level of education, n (%) (n=84)	
College/university	52 (61.90)
High school	27 (32.14)
Intermediate school	3 (3.57)
Primary school	2 (2.38)
Current position, n (%) (n=84)	
Housewife	51 (60.71)
Employed	21 (25.00)
Student	12 (14.29)
Relationship with partner spouse/partner, n (%) (n=84)	
Married and living together	81 (96.43)
Married and not living together	2 (2.38)
Widowed / separated / divorced	1 (1.19)
Patients’ reproductive history	
Pregnancy-n, n (%) (n=84)	
0-3	64 (79.01)
4-6	14 (17.28)
≥7	3 (3.70)
Plan on having more children, n (%) (n=84)	
Yes	49 (58.33)
No	11 (13.10)
Maybe	24 (28.57)
Contraceptive method ever used, n (%) (n=84)	
Oral contraceptives	42 (50.00)
Implanon Implant (previous use)	34 (40.48)
Natural methods, e.g., withdrawal	24 (28.57)
Male condoms	19 (21.6)
Intrauterine devices (IUD)	14 (16.67)
Three months injectable	5 (5.95)
Female condoms	1 (1.19)
None	8 (9.52)

Thirty-five (41.67%) women heard about Implanon from clinic staff and approximately one-third of the women (28, 33.33%) heard about it from social media. Fifty-five (65.48%) women chose to use the implant because of its long duration of action. Participants were asked whether they received counseling before Implanon was implanted. Most participants (75, 89.29%) reported being counseled on how long Implanon lasts, and 62 (73.81%) were counseled on possible side effects. However, 47 (55.95%) did not receive information about when to return for follow-up. Forty (47.62%) women had used an Implanon implant for ≤1 year and 23 (27.38%) women had used it for three years.

When asked if they had removed Implanon, 46 (54.76%) had not and 28 (33.33%) had removed it within the year prior to the survey. The women were asked about the reasons that made them remove Implanon or what would compel them to remove it if it had not been. Thirty-six (42.86%) said they would remove it when the three years are completed, and 29 (34.52%) said they would remove it due to a desire to conceive.

Regarding side effects of Implanon use, 46 (54.76%) women reported weight gain and 33 (39.29%) women mentioned menstrual irregularities. Other side effects experienced were headaches in 31 (36.90%) women and pain/numbness in the arm in 24 (28.57%). 

When they were asked if they were willing to have Implanon inserted in the future, 34 (40.48%) said that they were willing and 22 (26.19%) said that they were not willing. Furthermore, 48 (57.14%) participants said they would recommend the implant to their family and friends (Table 2).

**Table 2:** Data on Implanon®* use, removal, and side effects *Organon & Co., Jersey City, New Jersey, United States

When was your Implanon implant inserted? n (%) (n=84)	
3 months ago	16 (19.05)
6 months ago	14 (16.67)
9 months ago	6 (7.14)
1 year ago	18 (21.43)
2 years ago	9 (10.71)
3 years ago	12 (14.29)
> 3 years ago	9 (10.71)
How long have you had Implanon implant? n (%) (n=84)	
< 6 months	18 (21.43)
6 months -1 year	22 (26.19)
>1 year, < 2 years	11 (13.10)
2 years	6 (7.14)
3 years	23 (27.38)
>3 years	4 (4.76)
When have you removed Implanon (if removed)? n (%) (n=84)	
3 months ago	13 (15.48)
6 months ago	3 (3.57)
9 months ago	5 (5.95)
1 year ago	7 (8.33)
2 years ago	2 (2.38)
3 years ago	6 (7.14)
> 3 years ago	2 (2.38)
Implant not removed yet	46 (54.76)
How did you hear about Implanon for the first time? n (%) (n=84)	
Clinic staff	35 (41.67)
Social media	28 (33.33)
Friend	20 (23.81)
Relative	10 (11.90)
Poster / Pamphlet	7 (8.33)
Television / Radio	1 (1.19)
Can't remember	3 (3.57)
Why did you decide to use the Implanon Implant? n (%) (n=84)	
Wanted to use a long-acting method	55 (65.48)
Was having side effects from other methods	25 (29.76)
Healthcare worker recommended	23 (27.38)
A friend/relative recommended	14 (16.67)
Did not want to return to the clinic frequently for follow up	7 (8.33)
Did you receive information/counselling on the following before inserting the Implanon implant? n (%) (n=84)	
How long does it last	75 (89.29)
Possible side effects	62 (73.81)
When to come for removal	60 (71.43)
Advantages	52 (61.90)
How it works to prevent pregnancy	50 (59.52)
When to come for follow up	37 (44.05)
Side effects experienced while using Implanon n (%) (n=84)	
Weight gain	46 (54.76)
Menstrual irregularities	33 (39.29)
Headaches	31 (36.90)
Pain/numbness in the arm	24 (28.57)
Loss of libido	22 (26.19)
Dizziness	12 (14.29)
Weight loss	7 (8.33)
Reasons for removal of Implanon (if they removed it or would like to remove it), n (%) (n=84)	
Expired/completed 3-year duration	36 (42.86)
Weight gain	31 (36.90)
Want to conceive	29 (34.52)
Menstrual irregularities	24 (28.57)
Headaches	19 (22.62)
Loss of libido	13 (15.48)
Pain/numbness in the arm	13 (15.48)
Dizziness	12 (14.29)
Partner/Husband Request	5 (5.95)
No current partner	4 (4.76)
Weight loss	4 (4.76)
Pregnancy occurred	3 (3.57)
Infection at the site of the implant	2 (2.38)
Other family member wants me to remove it	1 (1.19)
Willing to have an Implanon implant in the future, n (%) (n=84)	
Yes	34 (40.48)
No	22 (26.19)
Maybe	22 (26.19)
Do not know	6 (7.14)
Recommend the implant to family and friends, n (%) (n=84)	
Yes	48 (57.14)
No	16 (16.67)
Maybe	17 (20.24)
Do not know	5 (5.95)

Bleeding patterns varied among women. Twenty (23.81%) women reported having a spotting pattern of bleeding, and 24 (28.57%) reported irregular bleeding. Only 10 (11.90%) women reported regular bleeding patterns, and 30 (35.71%) said that no bleeding occurred during Implanon use. Of those who reported any type of bleeding, 27 (50.00%) women experienced prolonged bleeding. Fifty-three (63.10%) women experienced a bleeding pattern abnormal to them (Table 3).

**Table 3:** Bleeding patterns of the women on Implanon®* *Organon & Co., Jersey City, New Jersey, United States

Bleeding Pattern	
Was the bleeding pattern normal to you? n (%) (n=84)	
No	53 (63.10)
Yes	31 (36.90)
Regularity, n (%) (n=84)	
No bleeding	30 (35.71)
Irregular	24 (28.57)
Spotting	20 (23.81)
Regular	10 (11.90)
Volume, n (%) (n=54)	
Normal	22 (40.74)
Too little	18 (33.33)
Too much	11 (20.37)
Undetermined	3 (5.56)
Frequency, n (%) (n=54)	
Infrequent	24 (44.44)
Normal	12 (22.22)
Too often	10 (18.52)
Undetermined	8 (14.81)
Duration, n (%) (n=54)	
Too long	27 (50.00)
Normal	17 (31.48)
Too short	4 (7.41)
Undetermined	6 (11.11)

**Figure 1:**
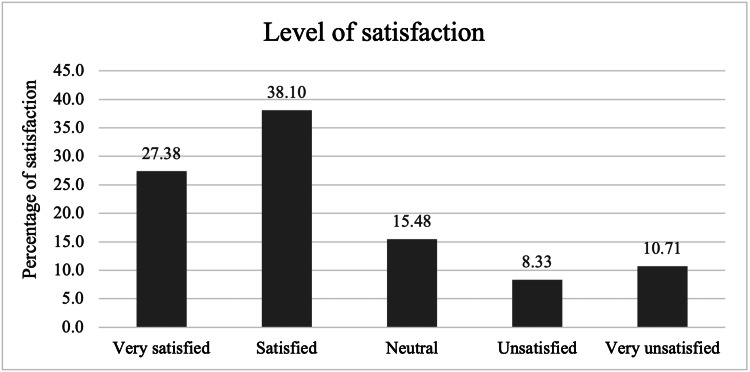
Satisfaction of women with the use of Implanon®* *Organon & Co., Jersey City, New Jersey, United States

When the women were asked how satisfied they were with Implanon, 23 (27.38%) were very satisfied, 32 (38.10%) were satisfied, 13 (15.48%) were neutral, seven (8.33%) were unsatisfied, and nine (10.71%) were very unsatisfied (Figure 1).

## Discussion

As previously mentioned, there is a lack of information about Implanon use in Saudi Arabia. As such, this study identified women’s overall satisfaction with the use of the subdermal contraceptive Implanon, the average duration of its use, and the most common factors associated with its removal at SPC-PHC. Regarding satisfaction with Implanon's use, 65.48% of women were satisfied and 19.04% were unsatisfied. These results indicate high overall satisfaction with Implanon use, which is comparable to other studies performed in different countries, such as a study conducted in Switzerland, which found that 79% of women were satisfied [8]. Other studies conducted in Brazil and St. Louis, Missouri, found overall satisfaction rates of 90% and 79%, respectively [8,17,14].

In a survey conducted by Beesham et al., the most common cause of removal was the end of the three-year lifespan of the implant (75.9%) [16]. In the current study, the most commonly reported cause of removal was also the end of the three-year duration (42.86%); however, this percentage was lower than what Beesham et al. reported, which could be attributed to the difference in sample size. In the current study, the age group with the most participants was 25-30 years (40.4%). Similarly, a study carried out at the Atrium Medical Center in the Netherlands that included 214 women found that women between 25 and 29 years of age were in the plurality (23%) [18]. Furthermore, the latter study found that most of the women who chose the implant (90%) had had three or fewer pregnancies, which is in line with what was found in this study [18].

The findings of this research revealed that the most common reason for starting Implanon was its prolonged duration of action, which was also the most common reason for choosing Implanon in a study in Upper Egypt [13]. The data suggest that most study participants in this current research received counseling on using Implanon, including its advantages, course of action, and side effects. In contrast, a study conducted in Kinshasa found that most women received low-quality counseling, which affected the continuation rate [19].

In a study conducted in Brazil, Implanon's most common side effect was weight gain, which is comparable to our findings [17]. In contrast, in the study conducted in Kinshasa, the most common side effect was bleeding [19]. Our data indicate that the most common reason for discontinuing Implanon was the end of the three-year lifespan unlike studies conducted in Brazil and Kinshasa, in which the most common reason was side effects [17,19].

The most commonly reported bleeding pattern with Implanon was amenorrhea, which was also the case in the study conducted in Switzerland [8]. Furthermore, most of those with bleeding reported that bleeding was infrequent and with a longer duration, which coincides with the results in a report by Dionne et al., which stated that the most common side effect and reason for removal was infrequent bleeding [3]. The findings suggest that most participants were unsatisfied with their bleeding pattern, which contradicts the results of the Swiss study, which found that most women were satisfied with their bleeding pattern on Implanon [8].

This research provides valuable insight into the general satisfaction with contraceptive implants as, to our knowledge, there are no other studies focused on this contraceptive method in Saudi Arabia. The results point to a high level of satisfaction with this contraceptive, despite its side effects, which include weight gain, headaches, and arm numbness. The counseling that all the women received before insertion could explain the high satisfaction rate. Furthermore, over half of the women were under 30, and most of the women in this study had heard about Implanon through social media, which could explain the high acceptance rate among young women. Most of the women had used oral contraceptives before Implanon. However, most oral contraceptives require daily use, and adherence is necessary unless a woman wishes to become pregnant. Notably, the most common reason for inserting Implanon was the prolonged duration of action and the reduced need for follow-up.

This study has some limitations. This study was cross-sectional, so the women were not followed up and not all of the women who were selected had completed their course of Implanon; thus, they could not be assessed for any new or reoccurring side effects. Further, a selection bias may be observed, as the mobile survey was sent to women treated within a single center; thus, the results cannot be generalized to other populations. The response rate was low, perhaps because only MNGHA personnel and their families can be treated at the SPC-PHC, thus, decreasing our overall sample size. The low response rate may also be explained by some women providing a family member's contact information instead of their own, resulting in the survey being sent to a woman's family member instead of herself.

## Conclusions

Most women who were provided Implanon at the SPC-PHC are satisfied with its use. Moreover, most users will remove the implant due to reaching the end of the three-year lifespan rather than due to side effects. Furthermore, most women would recommend using Implanon to their family and friends. The most common side effect reported after insertion was weight gain, followed by headaches. We recommend that Implanon be provided in other healthcare centers due to its ease of use, long duration of action, and high level of satisfaction. We also recommend that studies be performed on a larger population. Further studies on Implanon are necessary, as the current literature does not discuss the use of this contraceptive method in Saudi Arabia.
